# Secondary Structure and Subunit Composition of Soy Protein* In Vitro* Digested by Pepsin and Its Relation with Digestibility

**DOI:** 10.1155/2016/5498639

**Published:** 2016-05-19

**Authors:** Yong Yang, Zhongjiang Wang, Rui Wang, Xiaonan Sui, Baokun Qi, Feifei Han, Yang Li, Lianzhou Jiang

**Affiliations:** ^1^Key Laboratory of Processing Agricultural Products of Heilongjiang Province, College of Food and Bioengineering, Qiqihar University, Qiqihar, Heilongjiang 161006, China; ^2^College of Food Science, Northeast Agricultural University, Harbin, Heilongjiang 150030, China

## Abstract

In the present study,* in vitro* digestibility and structure of soybean protein isolates (SPIs) prepared from five soybean varieties were investigated in simulated gastric fluid (SGF), using FT-IR microspectroscopy and SDS-PAGE. The result indicated that *β*-conformations were prone to be hydrolyzed by pepsin preferentially and transformed to unordered structure during* in vitro* digestion, followed by the digestion of *α*-helix and unordered structure. A negative linear correlation coefficient was found between the *β*-conformation contents of five SPIs and their* in vitro* digestibility values. The intensities of the protein bands corresponding to 7S and 11S fractions were decreased and many peptide bands appeared at 11~15 kDa during enzymatic hydrolysis. *β*-conglycinin was poorly hydrolyzed with pepsin, especially the *β*-7S subunit. On the other hand, basic polypeptides of glycinin degraded slower than acidic polypeptides and represented a large proportion of the residual protein after digestion. 11S-A_3_ of all SPIs disappeared after 1 h digestion. Moreover, a significant negative linear correlation coefficient (*r* = −0.89) was found between the *β*-7S contents of five SPIs and their* in vitro* digestibility values. These results are useful for further studies of the functional properties and bioactive properties of these varieties and laid theoretical foundations for the development of the specific functional soy protein isolate.

## 1. Introduction

Soybean protein isolate (SPI) is the main by-product of producing soybean oils, which have been widely used in many protein-based food formulations because of their excellent nutritional and functional properties, availability, and low cost [1–3].

In the past few years, there has been an increasing interest in the structural design of food-based delivery systems to encapsulate, protect, and release bioactive components believed to benefit human health [4]. However, low digestibility of plant proteins, together with a limiting content of essential amino acids (methionine, cysteine, and tryptophan), represents a major issue for their low nutritional value compared with animal proteins [5]. The digestion rate of proteins is known to be the main determinant in their assimilation in the diet, especially for elderly people, who are prone to sarcopenia [6, 7]. Moreover, undigested proteins entering the colon are suspected of favoring carcinogenesis [8]. Gastric digestion by pepsin, which takes place before the trypsin/chymotrypsin reactions, affects protein digestibility [9]. Recently, increasing evidences suggest that (1) the structural properties of proteins in plant foods have a major role in resistance to denaturation [10, 11] and (2) plant proteins manifest stability upon gastrointestinal digestion [12, 13]. However, few studies have been performed on the relationship between subunits composition and structure of protein and digestibility [14]. Higher proportions of the aromatic and basic amino acids were found in the digestion products compared to those in the intact protein sources, reflecting the specificity of the proteolytic enzymes [15]. Wang et al. [16] reported that hemp (*Cannabis sativa* L.) protein constituents of edestin were rapidly digested by pepsin, to release oligopeptides with molecular weight (MW) less than 10.0 kDa and suggested that the* in vitro* digestibility of HPIs was comparable to that of SPI. Sze-Tao and Sathe [17] suggested that pepsin hydrolysis of almond (*Prunus dulcis* L.) protein isolate initially produced polypeptides in the MW range 15–36 kDa followed by several small MW polypeptides (15–20 kDa) and some in MW range 20–40 kDa. Ortiz and Wagner [18] reported that the enzymatic hydrolysis of soy protein produced a decrease in the high-molecular-mass aggregates, a loss of *α*′- and *α*-7S subunits, and the disappearance of bands corresponding to high-molecular-weight peptides with the appearance of 43 and 20–30 kDa bands during hydrolysis. Carbonaro et al. [5] reported a strong inverse correlation between the relative spectral weights of the *β*-sheet structures and* in vitro* protein digestibility values. In principle,* in vitro* digestion models provide a useful alternative to animal and human models by rapidly screening food ingredients [19].

Different soybean varieties presented different protein structures and properties, which endow soy protein with different bioactivity and functionality, which may further affect its applications in the food industry [2, 3, 20]. In the present study, five soybean varieties selected for evaluation are widely grown in China. Protein isolates were prepared from the seeds and their composition and molecular structures were studied by FT-IR microspectroscopy and SDS-PAGE electrophoresis. Furthermore, the relationships between protein composition and molecular structure of food proteins and their digestibility were considered and discussed. The results will be useful for further studies of functional properties as well as bioactive properties of these varieties and laid theoretical foundations for the development of the specific functional soy protein isolate.

## 2. Materials and Methods

### 2.1. Materials and Chemicals

Five varieties of soybeans, namely, Dongnong46 (A), Hedou21 (B), Shandou125 (C), Diandou4 (D), and Jingdou15 (E), were donated by the Key Laboratory of Soybean Biology of Education Ministry, Soybean Research Centre of Northeast Agriculture University, China. Pepsin was purchased from Solarbio (Beijing, China); the activity of this commercial pepsin activity was found to be 3000 ± 50 units/mg using haemoglobin as substrate. All other chemicals used in the present study were of analytical grade.

### 2.2. Preparation of Soy Protein Isolates (SPIs)

Soybeans were ground to flour in a Wiley mill equipped with a 60-mesh screen. Flours were defatted three times with hexane (1 : 6, w/v) and air-dried 24 h. Defatted flours were dissolved in 1 : 10 ratio (w/v) in deionized water and shaken for 1.5 h at 45°C with the pH adjusted to 8.0. The slurry was then centrifuged at 10000 g at 4°C for 30 min (Beckman Coulter, Model Allegra 64R, Fullerton, CA, USA). The collected supernatants were subjected to acid precipitation by adjusting pH to 4.5 using 2 M HCl. After centrifugation (6000 g, 4°C, 30 min), the recovered precipitates obtained were resolubilized in deionized water at pH 7, then dialyzed, and freeze-dried. All the five varieties of SPIs showed protein contents ranging from 86.67% to 90.77% (dry basis). SPIs prepared by five varieties of soybeans were labeled as SPI-A (Dongnong46), SPI-B (Hedou21), SPI-C (Shandou125), SPI-D (Diandou4), and SPI-E (Jingdou15), and the main compositions of five varieties of SPIs were listed in Table 1.

### 2.3. *In Vitro* Pepsin Digestion

Pepsin solution was prepared freshly for each assay by dissolving pepsin in the simulated gastric fluid (SGF) by vortexing for 5 min and the resulting solution was placed on ice. The concentrations of all the test samples were 5% w/w of the SPIs for SGF digestion assay. SGF was prepared according to the standard procedures described by the United States Pharmacopoeia (USP, 32nd edition) and consisted of 3.2 mg/mL purified pepsin (800–3000 units/mg protein, pH 1.2), containing 2.0 mg/mL NaCl [21, 22]. The* in vitro* gastric model consisted of a conical flask (100 mL) containing 10 mL of SGF-pepsin maintained at 37°C with continuous shaking at 95 rpm/min in a temperature-controlled water bath. The SGF-pepsin solution was preincubated for 5 min, followed by addition of 10 mL of diluted SPI solutions. The ratio of pepsin to SPI was 1 : 100 on a weight basis [23]. Aliquots (10 mL) were withdrawn into beakers at intervals (0, 0.25, 0.5, 1, 2, 3, 4, and 5 h) during the incubation at 37°C, and 75 *μ*L of 200 mM Na_2_CO_3_ (pH 11.0) was added to each mixture to stop the reaction by neutralisation. The digestion was replicated in triplicate.

### 2.4. Degree of Hydrolysis (DH)

DH of SPI digests was analyzed using the OPA method. A usual way of monitoring the extent of protein degradation during the course of the hydrolysis reaction is obtaining the percentage of the total number of peptide bonds in a protein cleaved during the hydrolysis reaction or DH [24]. The OPA reagent was prepared as follows: Disodium tetraborate decahydrate (7.62 g) and 200 mg sodium dodecyl sulphate (SDS) were dissolved completely in 150 mL of deionized water. OPA (160 mg) was dissolved in 4 mL of ethanol and then transferred to the above-mentioned solution. 176 mg of DTT was added to the solution and made up to 200 mL with deionized water. The serine standard was prepared as follows: 50 mg serine was diluted in 500 mL deionized water (0.9516 meqv/L). Diluted SPI digests (400 *μ*L) were mixed with 3 mL OPA reagents for exactly 2 min, and the absorbance (340 nm) was measured by SP-721 UV spectrophotometer (Shanghai, China). Free amino groups in SPI digests were expressed as serine amino equivalents (serine NH_2_ equv) [25]. We calculated DH with the following equations:

(1) DH%=hhtot×100,h=Serine  NH2−βα,

where *β* equals 0.342 mequv/g, *α* equals 0.970, and *h*_tot_ equals 7.8 mequv/g for soy protein.

### 2.5. FT-IR Microspectroscopy

FT-IR absorption spectra from 4,000 to 400 cm^−1^ were acquired in the transmission mode by Nicolet Magna IR 550 FT-IR spectrometer (Thermo Fisher Scientific Inc., Waltham, MA, USA) continuously purged with dry air and equipped with liquid nitrogen cooling MCT detector. Heated SPI samples were first freeze-dried and then produced by pressing in KBr windows (1.5 mg protein to 200 mg KBr) on a Carver press at 5-6 T pressure. The recording conditions for each FT-IR spectrum were as follows: 64 scans, a triangular apodization function, and a resolution of 4 cm^−1^. Each spectrum was obtained by coadding 256 interferograms at a spectrum resolution of 2.0 cm^−1^. The decomposition of amide I band was performed in the region of 1700–1600 cm^−1^. A second-derivative analysis (“peak fitting” procedure) of the IR-SD, which was shown previously to provide reliable quantitative information, was used to obtain quantitative analysis of the secondary structural components of SPI [26]. The “peak fitting” procedure was applied to the linear baseline correction, the Fourier self-deconvolution, and the deconvoluted (difference) spectrum to resolve and quantify its individual component bands according to a Gaussian curve fit (GCF) [26].

### 2.6. SDS-PAGE Electrophoresis

Sodium dodecyl sulphate-polyacrylamide gel electrophoresis was performed on a discontinuous buffered system with mercaptoethanol according to the method of Laemmli [27] using 15% separating gel and 4% stacking gel. Lyophilized SPI samples (2 mg mL^−1^ in buffer containing 0.0625 M Tris-HCl, 10% glycerin, 2% SDS, and 5% 2-mercaptoethanol, 0.0025% bromophenol blue) were incubated for 1 h at room temperature and then heated at 95°C for 5 min. Aliquots (15 *μ*L) of the prepared samples were loaded onto the gels. After the electrophoresis run, the gels were stained for 30 min with Coomassie Brilliant Blue R-250 (methanol-water-acetic acid (v/v), 5 : 4 : 1). Prior to destaining, the gels were washed extensively with deionized water and then destained with several volumes of the destaining solution comprised of 12% methanol and 7% acetic acid in deionized H_2_O over a period of at least 2 days [28, 29]. All images were saved as 300 DPI jpg files, and the densitometric analyses were measured for all the bands on the SDS-PAGE gels using the Glyko BandScan software (Version 5.0, Glyko Inc., Novato, CA, USA).

### 2.7. Statistical Analysis

All of the tests were performed in triplicate, and the results are given as means ± standard deviations. Duncan's multiple range test was used to evaluate significant differences (*P* < 0.05). Secondary structure elementsand DH were analyzed using the Pearson correlation coefficient; all significance tests were 2-tailed with significance considered as *P* < 0.05. The same statistical analysis was conducted for shrinkage factor corrected and noncorrected data. All statistical calculations were performed using a commercially available computer software package (SPSS software, Version 22).

## 3. Result and Discussion

### 3.1. Degree of Hydrolysis

**Figure 1:**
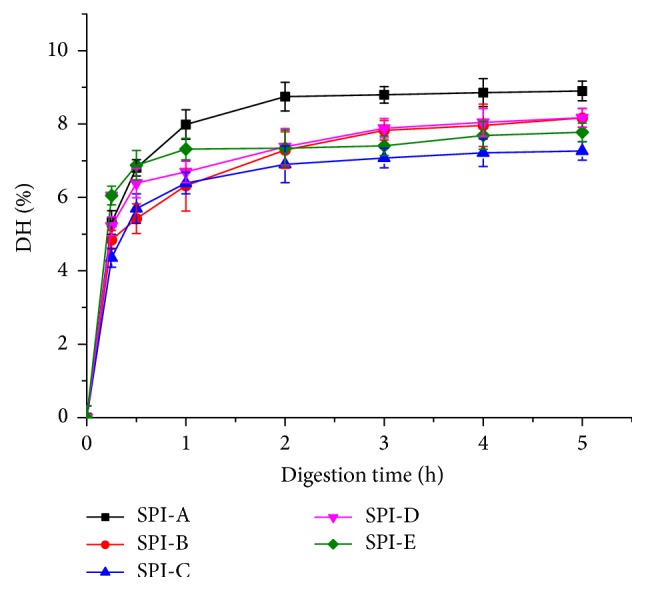
DH of SPIs with different digestion times.

The digestion time for each step (e.g., mouth, stomach, and small intestine) is an important factor to establish when designing an appropriate* in vitro* digestion model. In previous study a digestion time of 2 hours was used in many* in vitro* digestion models to simulate transit across stomach, small intestine, and large intestine [30–33]. However,* in vitro*, the digestion time depends upon individual characteristics (e.g., age, sex, health status, mental state, and time of day) and food properties (e.g., total amount, composition, and particle size) and may vary quite considerably [4]. Thus, DH of SPIs was recorded from 0 to 5 h in order to represent the digestion of different people by the* in vitro* model. As shown in Figure 1, DH of SPIs progressed rapidly at initial stage and then relatively slowly over time before reaching a plateau. This trend is typical for protease hydrolysis [34]. It should be noted that when 2 h digestion was introduced, SPI-A obtained the highest DH values (8.75), followed by SPI-D (7.37), SPI-E (7.33), SPI-B (7.28), and SPI-C (6.90). These results suggested that pepsin can access cleavage sites more easily for SPI-A compared to others; the structure of SPI-A was more easily accessible to enzymatic hydrolysis by pepsin. To gain additional information at a molecular level about the relationship between structure of food proteins and their digestibility, IR spectroscopy and SDS-PAGE electrophoresis were applied to examine the secondary and quaternary structure of proteins.

### 3.2. Assignments of Amide I Band Components to Distinct Secondary Structure Elements

**Figure 2:**
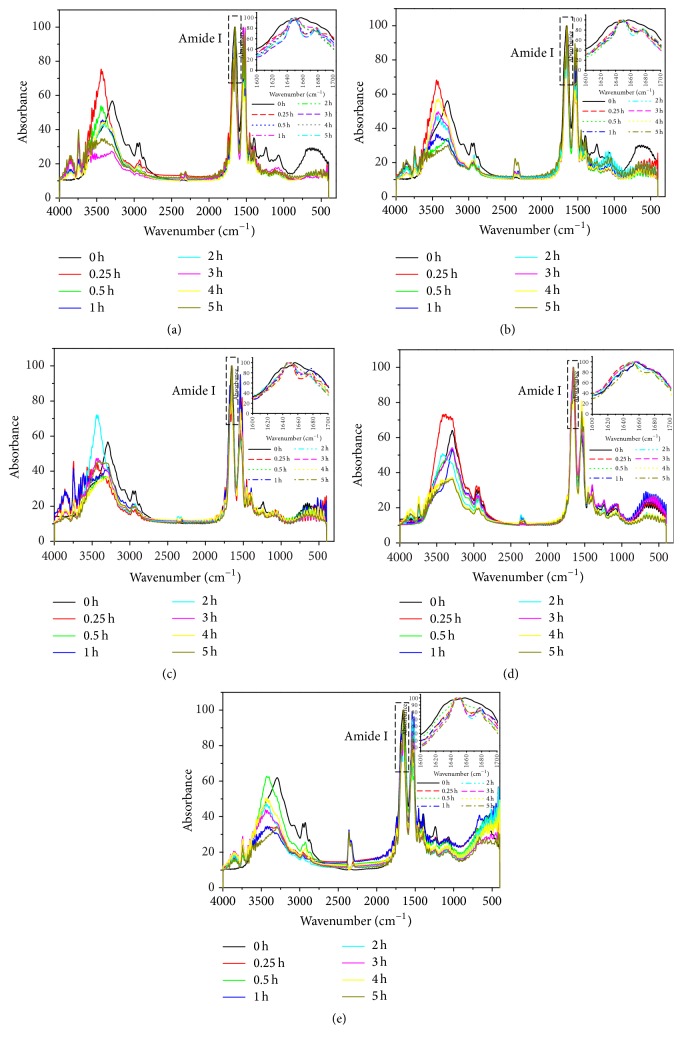
FT-IR spectra of SPIs with different digestion times: (a) SPI-A; (b) SPI-B; (c) SPI-C; (d) SPI-D; and (e) SPI-E. Data are expressed as the mean of three replicates; values followed by different letters are significantly different at *P* < 0.05.

Figure 2 showed the FT-IR spectra of five varieties of SPIs, while amide I band had been marked. Amide I mode originates mainly from the C=O stretching vibration of the polypeptide backbone [35]. The major factors responsible for the conformational specificity of amide I band are its sensitivity to hydrogen bonding and the characteristic coupling between transition dipoles, the latter leading to characteristic splitting effects. The magnitude of this splitting depends on the orientation and distances of interacting dipoles and thus provides information about the geometrical arrangements of peptide groups in a polypeptide chain.

The quantitative analysis of secondary structural components of proteins can be obtained by various experimental methods. Analysis of the second-derivative of the IR-SD was shown previously to provide reliable quantitative information [36]. The areas of assigned amide I bands in the second-derivative spectra correspond linearly to the amount of the different types of secondary structures present in the protein. In this study, prior to the second-derivative analysis, a baseline adjustment was performed to accurately measure the band areas of second-derivative spectra in amide I and further study Fourier self-deconvolution (FSD), which can substantially influence the number, position, and intensity of the bands resolved by a Gaussian curve fit (GCF) [36, 37].

**Figure 3:**
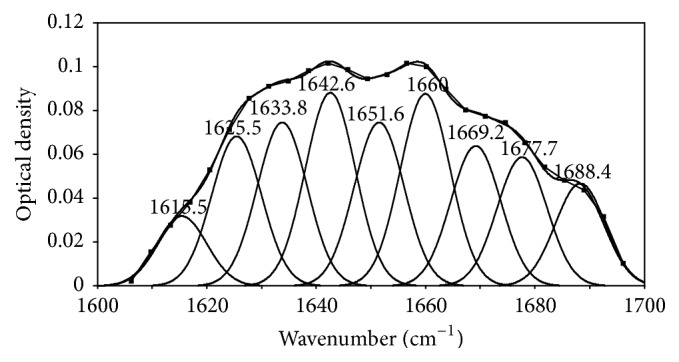
Deconvolution of amide I spectra (continuous curve), the GCF bands thereof (point line), and the second-derivative spectra of SPI.

As shown in Figure 3, FSD was firstly used to obtain deconvoluted spectra in this study, while the key meaningfulness of the FSD method is to select the conditions that achieve the maximum band narrowing and keeping the increase in noise and the appearance of side-lobes at minimum. Second-derivative analysis was used to separate bands, and second-derivative spectra allow the identification of various secondary structures present in the protein. Detailed information could be obtained by analyzing the deconvoluted spectra. The GCF was adjusted to give the best least squares fit of the individual bands to each deconvoluted spectrum, followed by a second-derivative analysis [36].

**Figure 4:**
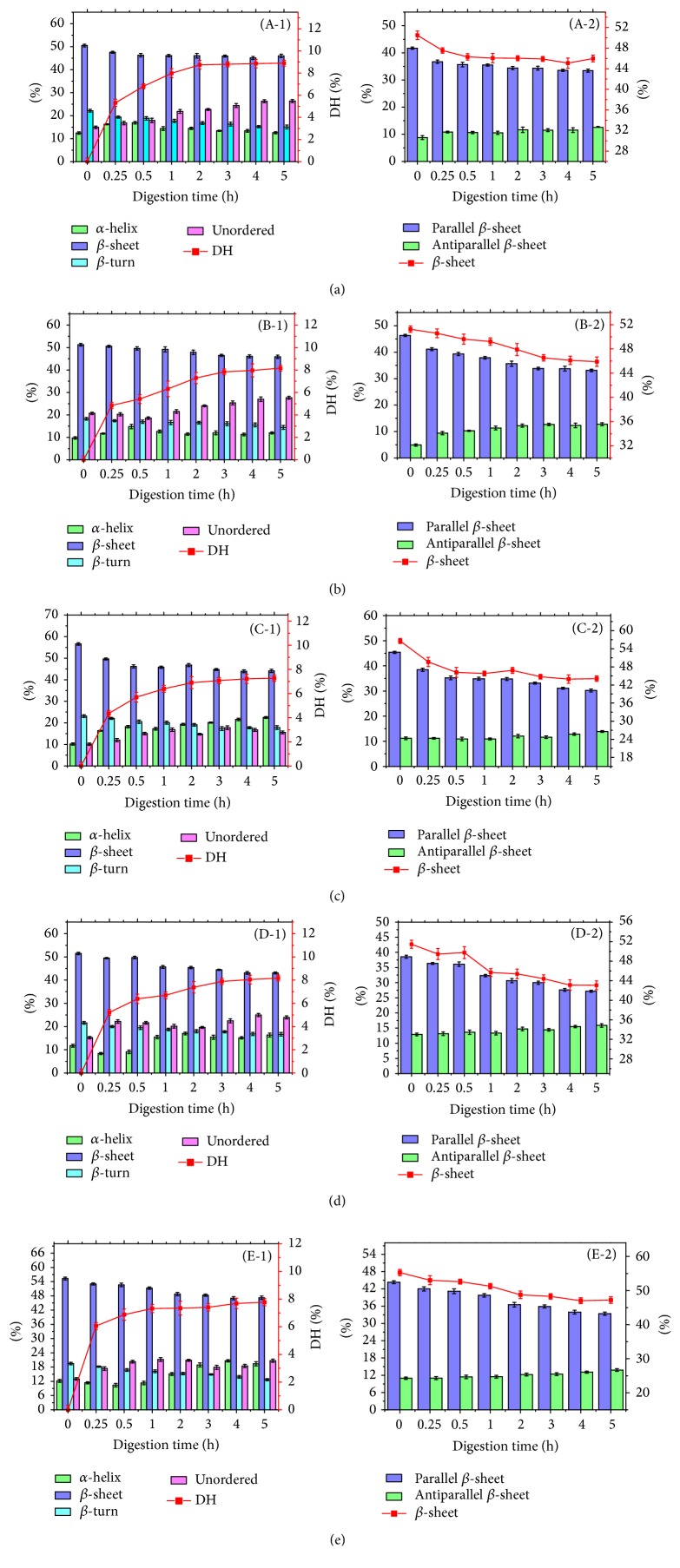
Amide I band components to distinct secondary structure elements of SPI with different digestion times: A-1 and A-2: SPI-A-1, SPI-A-2; B-1 and B-2: SPI-B-1, SPI-B-2; C-1 and C-2: SPI-C-1, SPI-C-2; D-1 and D-2: SPI-D-1, SPI-D-2; and E-1 and E-2: SPI-E-1, SPI-E-2.

Our second-derivative band positions followed with previous data from the literature that reported a strong band for *α*-helix with a frequency around 1650–1660 cm^−1^ [38]. We also obtained several bands corresponding to *β*-sheet in the frequency region of 1618–1640 cm^−1^ and 1670–1690 cm^−1^ [39]. The fact that *β*-sheets in proteins appear as parallel or antiparallel strands, in the frequency region of 1618–1640 cm^−1^ corresponding to parallel *β*-sheet, while those in the range of 1670~1690 cm^−1^ originate from antiparallel *β*-sheet structures [5, 35, 40]. A series of bands corresponding to *β*-turn appeared in the 1660–1670 cm^−1^ range [39, 41]. The random coil structure had a strong band in the 1640–1650 cm^−1^ range [38]. The percentages of *α*-helix and *β*-sheet and unordered and *β*-turn secondary structures in SPI are shown in Figure 4.

In this study, the second-derivative and deconvoluted spectra of five varieties of SPIs predominately indicated *β*-sheet, which is similar to previous studies. Herrero et al. [42] reported that SPI contained 23.8% *α*-helix, 43.6% *β*-sheet, 19.7% turns, and 13.0% unordered structures by Raman spectroscopy. Similarly, a midinfrared study of SPI reported that its secondary structure was 28.4% *α*-helix, 49.7% *β*-sheet, and 20.9% *β*-turns [43]. Yu et al. indicated higher *β*-sheet (55.2%) and lower *α*-helix (14.4%) contents than those observed in the present study [44]. Zhao et al. [38] reported 17.0% *α*-helix, 47.3% *β*-sheet, 35.9% turns, and no unordered structures for 11S globulin in aqueous buffer by FT-IR spectroscopy and 16.5% *α*-helix, 46.7% *β*-sheet, 35.9% turn, and no unordered structures for 7S globulin. In comparison, the content of secondary structural elements differed in varieties. SPI-A had the highest content of *β*-sheet structure but lowest *α*-helix structure content and SPI-B showed the highest unordered structure content and lowest *α*-helix content, while SPI-C had the highest *β*-sheet and *β*-turn structure content but the lowest unordered structure content.

Bamdad et al. [45] reported that most of the secondary structures of barley hordein did not change after short periods of pepsin hydrolysis. After 3 h of pepsin hydrolysis, the content of both *β*-turn and random coils decreased significantly, and two major peaks at 1617 and 1610 cm^−1^ appeared that correspond to intermolecular *β*-sheet and amino acid side chain residues. Zhao et al. [46] reported that hydrolysis caused a pronounced loss of ordered structures, especially *α*-helixes; a small percentage of *β*-turns was transformed into *β*-sheets.

However, in this study, with the increasing digestion time, *β*-sheet and *β*-turn structures of five SPIs decreased. The decreased *β*-sheet of those SPIs may be attributed to the loss of parallel *β*-sheet, while the amount of the antiparallel *β*-sheet increased slightly. With the increasing digestion time, the percentage of *α*-helix appeared to increase in some samples, from time 0 to 5 hours, although not steadily. With the increasing digestion time, the percentage of unordered structure of most SPIs increased. The digestion of *α*-helix and *β*-conformation may increase the content of unordered structure. Thus, it can hardly determine the digestion of unordered structure of SPIs, but the decreased unordered structure content of SPI-B at initial time and SPI-C at 2 h may be related to the digestion of unordered structure.

Generally, soy protein structures tend to unfold to expose more *β*-conformation in SGF increasing the accessibility of susceptible sites to pepsin; it can be speculated from the digested time of each structure (when the content of four structure decreased) that exposed *β*-conformations were prone to be hydrolyzed by pepsin preferentially and transformed to unordered structure during* in vitro* digestion, followed by the digestion of *α*-helix and unordered structure.

### 3.3. Relationship between Secondary Structure and Degree of Hydrolysis

Correlation analysis was used to determine the relationship between secondary structural elements and degree of hydrolysis; a significant negative linear correlation coefficient (*r* = −0.91) was found between the *β*-sheet contents of five SPIs and their* in vitro* digestibility values (2 h digestion time). Carbonaro et al. [5] also found a high negative linear correlation coefficient between the *β*-sheet contents of all proteins and the food digestibility values, and an inverse linear correlation was observed between antiparallel *β*-sheet structure and protein digestibility.

It is suggested that soy proteins with less *β*-sheet content have a better digestibility. The *β*-sheet structures are mainly stabilized by hydrogen bonds between the carbonyl groups (–CO) and the amino groups (–NH) and tend to be deeply buried in the polypeptide chain [47, 48]. Lee et al. [49] also reported that the involvement of *β*-sheets in the secondary structure of protein aggregates might be attributed to the relatively large surface areas for ordered hydrogen bonding. It can be deduced that the buried structural characteristic of *β*-sheet increases the compactness of protein structure, which is hard to unfold in gastric fluid, which reduces the accessibility of susceptible sites to pepsin. Tang and Sun [50] suggested that protein with higher random coil and concomitantly lower *β*-sheet contents had a more unordered and less compacted protein molecular structure, which is easy to be hydrolyzed. Besides, Carbonaro et al. [5] attributed the decrease in protein digestibility as a function of the amount of *β*-sheet conformations to its high hydrophobic character. In our previous study, we found surface hydrophobicity of soy protein increased with *β*-sheet content [26]. High hydrophobicity has been suggested to adversely affect the solubility of legume proteins by promoting protein-protein interaction and aggregate formation, reducing accessibility of susceptible sites to proteases [51].

As shown in Table 2, significant negative linear correlation coefficient was found between *β*-turn structure of five SPIs and DH as a function of time. The correlation coefficients of SPI-A, SPI-B, SPI-C, SPI-D, and SPI-E were −0.94, −0.86, −0.88, −0.91, and −0.78, respectively. The *β*-sheet structures of five SPIs were negative related to DH; the correlation coefficients of SPI-A, SPI-B, SPI-C, SPI-D, and SPI-E were −0.98, −0.86, −0.98, −0.84, and −0.76, respectively. It can be deduced that the increase of DH of soy protein is related to the loss of *β*-conformations during* in vitro* digestion, which indicates that enzymatic hydrolysis of *β*-conformations by pepsin promotes* in vitro* digestion of soy protein.

Moreover, similar significant negative correlation was also observed between parallel *β*-sheet structure of five SPIs and DH; the correlation coefficients of SPI-A, SPI-B, SPI-C, SPI-D, and SPI-E were −0.99, −0.98, −0.97, −0.83, and −0.73, respectively. In comparison, the contents of both *β*-sheet structure and parallel *β*-sheet structure of SPIs were negative related to DH, which suggested that* in vitro* digestion of SPIs decreased the *β*-sheet structure mainly attributed to the decreased parallel *β*-sheet structure. In addition, antiparallel *β*-sheet structures of SPI-A, SPI-B, and SPI-D were found to be positively related to DH, while no significant relation was observed between antiparallel *β*-sheet structures of SPI-C and SPI-E and their DHs. It can be deduced that parallel *β*-sheet structures of SPIs are more easily digested compared to antiparallel *β*-sheet.

No significant relation was found between the contents of *α*-helix of SPIs and DH except SPI-C. The content of random coil of SPI-A was found to be positive related to its DH, while no significant relation was observed between the other four SPIs and their DH. It can thus be seen that random coil and *α*-helix structure gradually formed during digestion of these SPIs.

### 3.4. SDS-PAGE Analysis

**Figure 5:**
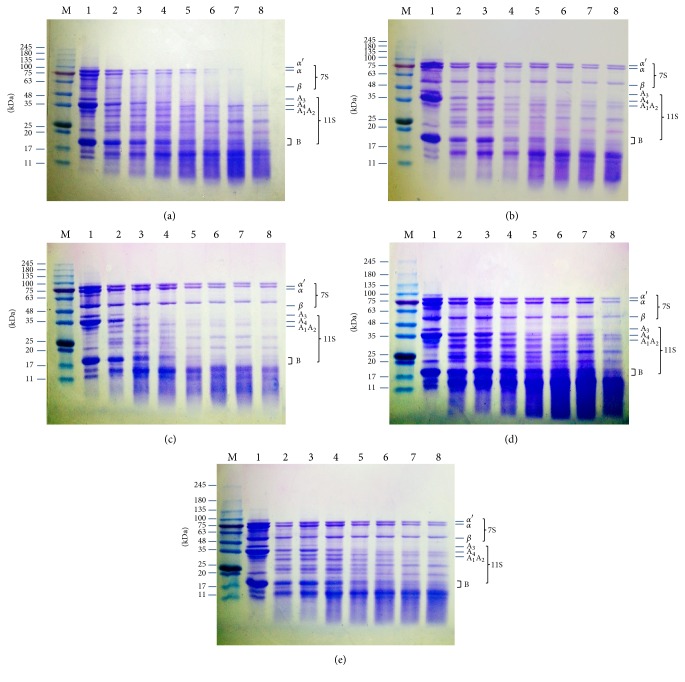
SDS-PAGE profile of different digestion times from five SPIS: (a) SPI-A; (b) SPI-B; (c) SPI-C; (D) SPI-D; and (e) SPI-E.

Figure 5 showed SDS-PAGE profiles of SPIs treated with pepsin for 0~5 h of digestion. SPIs profile showed typical protein bands belonging to 7S fraction (*α*, *α*′, and *β* subunits) and 11S fraction (acid (A) and basic (B) subunit). The molecular weights of the *α*′, *α*, and *β* subunits of the 7S fraction were approximately 80, 75, and 50 kDa, respectively. The subunit with the molecular weight of 36 kDa is acidic A_3_ polypeptide and the group of polypeptides around 34 kDa is a major group of acidic polypeptides (A_1_, A_2_, and A_4_). The cluster of protein bands with molecular weights of approximately 15 kDa are basic components of the 11S fraction. These results are consistent with earlier reports [52].

During pepsin digestion, the intensities of the protein bands corresponding to 7S and 11S fractions were decreased and many peptide bands appeared at 11~15 kDa indicating protein hydrolysis. The enzymatic hydrolysis produced a loss of *α*′-, *α*, and *β*-7S subunits and total disappearance of bands corresponding to these subunits of 7S of SPI-A after 3 h digestion. The enzymatic hydrolysis also led to gradual breakdown of acid and basic subunits of 11S globulin of SPI-A, in a hydrolysis time dependent manner. Amigo-Benavent et al. [53] reported that the electrophoretic bands corresponding to *α* and *β* subunits of 7S were observed whereas *α*′ subunit was not detected in the corresponding gastric digest.

The intensity of the bands corresponding to *α*′-, *α*-, and *β*-7S of SPI-B, SPI-C, SPI-D, and SPI-E decreased with time, together with the apparition of bands of A- and B-11S and the increased intensity of band of 11~15 kDa. Bands of A-11S of SPI-C mostly disappeared after 2 h of digestion.

Generally, basic polypeptides of glycinin degraded slower than acid polypeptides of five SPIs and represented a large proportion of the residual protein after digestion. Peng et al. suggested that the basic polypeptides of glycinin were more resistant to degradation than acidic polypeptides. This may be due to a difference in compactness of structure between polypeptides, basic polypeptides being more hydrophobic and thus more compact and less accessible to proteolytic enzymes [54]. Moreover, 11S-A_3_ of all five SPIs disappeared after 1 h digestion.

In comparison, the bands corresponding to *α*′-, *α*-, and *β*-7S of SPI-B, SPI-C, SPI-D, and SPI-E did not disappear after digestion, all of SPI-A being degraded by 3 h of digestion. Zhao et al. [55] suggested that *β*-conglycinin was poorly hydrolyzed with pepsin, consistent with the results of Astwood et al. [56]. Tsumura [57] also reported that glycinin fractions in native SPI disappeared with incubation at below pH 2.5; *β*-conglycinin fractions were not susceptible to peptic digestion at 37°C in the pH range of 1.5–2.5.

### 3.5. Relationship between Subunit Content of Soy Protein Isolates and Degree of Hydrolysis

As shown in Table 3, correlation analysis was used to determine the relationship between subunit contents and degree of hydrolysis; a significant negative linear correlation coefficient (*r* = −0.89) was found between the *β*-7S contents of five SPIs and its* in vitro* digestibility values (2 h digestion time), which suggested that SPI with low *β*-7S subunit content has a high DH. Zhao et al. suggested that pepsin could hydrolyze *α* and *α*′ subunits of *β*-conglycinin and A and B polypeptide chains of glycinin and *β* subunit of *β*-conglycinin could not be effectively hydrolyzed [55]. Amigo-Benavent et al. reported that *α* and *β* subunits of *β*-conglycinin glycoprotein partially survive the process of digestion [53]. Sadeghi et al. revealed that *α* and *α*′ subunits of *β*-conglycinin were digested completely within 2 h, whereas *β* subunit of *β*-conglycinin and the acidic and basic polypeptide components of glycinin were digested completely until 8, 12, and 48 h, respectively [58]. It can be deduced that the readily digestion of *β*-7S of SPI-A leads to a higher DH.

## 4. Conclusion

In summary, the* in vitro* digestibility and structure of soybean protein isolates (SPIs) prepared by five soybean varieties were investigated in simulated gastric fluid (SGF), using FT-IR microspectroscopy and SDS-PAGE, which indicated that the conformation of the protein might play a key role in resistance to proteolysis. In present study, *β*-conformations were prone to be hydrolyzed by pepsin preferentially during* in vitro* digestion, followed by the digestion of *α*-helix and unordered structure, while SPI with a lower *β*-conformation contents had a higher DH. *β*-conglycinin was more poorly hydrolyzed with pepsin compared to globulin, especially the *β*-7S subunit. Basic polypeptides of glycinin degraded slower than acid polypeptides, and in particular 11S-A_3_ of five SPIs all disappeared after 1 h digestion. A significant negative linear correlation coefficient (*r* = −0.89) was found between *β*-7S contents of five SPIs and their* in vitro* digestibility values.

## Figures and Tables

**Table 1:** Main compositions of five varieties of SPIs.

Sample	Protein content (%)	Oil content (%)	Moisture (%)	Ash (%)
SPI-A	86.67	0.61	5.11	4.55
SPI-B	89.64	0.48	5.15	3.64
SPI-C	88.63	0.82	5.23	4.33
SPI-D	90.17	0.44	5.08	4.36
SPI-E	90.77	0.49	5.13	3.36

**Table 2:** The correlation analysis between secondary structure and degree of hydrolysis.

Content of secondary structure	SPI-A	SPI-B	SPI-C	SPI-D	SPI-E
*α*-helix	0.08	0.38	0.95^*∗∗*^	0.51	0.41
*β*-turn	−0.94^*∗∗*^	−0.86^*∗∗*^	−0.88^*∗∗*^	−0.91^*∗∗*^	−0.78^*∗*^
Random coil	0.85^*∗*^	0.67	0.89	0.85	0.88
*β*-sheet	−0.98^*∗∗*^	−0.86^*∗∗*^	−0.98^*∗∗*^	−0.84^*∗∗*^	−0.77^*∗*^
Parallel *β*-sheet	−0.98^*∗∗*^	−0.98^*∗∗*^	−0.97^*∗∗*^	−0.83^*∗*^	−0.73^*∗*^
Antiparallel *β*-sheet	0.89^*∗∗*^	0.99^*∗∗*^	0.47	0.72^*∗*^	0.57

*∗* means significant correlation (*P* < 0.05).

*∗∗* means significant correlation (*P* < 0.01).

**Table 3:** The correlation analysis between protein subunit of soy protein isolates and degree of hydrolysis.

Sample		7S			11S	
		*α*′	*α*	*β*	Acidic	Basic
DH (2 h)	*r*	0.078	−0.175	−0.887^*∗*^	0.564	0.189

*∗* means significant correlation (*P* < 0.05).
